# Supplementary material for: Women’s experiences of maternal and newborn health care services and support systems in Buikwe District, Uganda: A qualitative study
Source: PLoS One. 2021 Dec 16;16(12):e0261414. doi: 10.1371/journal.pone.0261414 (PMC8675744; doi:10.1371/journal.pone.0261414)
Supplement: S1 File — (DOCX) [file pone.0261414.s001.docx]

**S1 File: Interview guide for key informants**

Introduction of the topic:

Many studies have been done with focus on delivery and breastfeeding. Most of these studies tend to exclude women with birth complications, even though they are more vulnerable and more likely to have problems with initiation of breastfeeding. This study will explore health care and support systems provided to mothers after giving birth, including those with birth complications, and you have been targeted because of your position as a health worker/TBA/Community leader.

I will first ask you a few introductory questions before going on to the topic of birth complications and breastfeeding.

Icebreaker questions:

- Can you tell me a bit about yourself?
- Can you tell me about your work?
- How long have you had this job?
- How will you describe a good day at work?

How will you describe a bad day?

Questions about delivery and birth:

1. Can you take me through the process of a normal delivery at your (work)place?
2. What is the first thing that happens after the baby is born?

- Where is the baby placed?
- Who takes care of the baby?

1. What happens next?

- Skin-to-skin?
- Cord clamping?
- Weighing?
- Breastfeeding?
- Mother care (sewing, placenta?)

1. What would you say are the most common complications when giving birth?
2. What happens to the baby after caesarean sections?

- Where is the baby placed?
- Who takes care of the baby?

Questions about breastfeeding:

1. When do you tell the mother to start breastfeeding?

- Do you provide her help with breastfeeding?
- How is the first milk?
- What do you tell the mother about this milk?

1. How is the baby fed if the mother is unable to?

- What advice are they given?

1. How do you care for preterm and low birth-weight babies (<2500 grams)?
2. Are you familiar with the Uganda Clinical Guidelines regarding care for newborns and breastfeeding?
3. Can you tell me about skin-to skin-contact (kangaroo care)?
4. When are mothers discharged from the hospital/clinic?

- What happens after discharge from the hospital?
- Where can the women seek help after discharge from the hospital?

1. Is there anything you would like to add?
