# Supplementary material for: Women’s experiences of maternal and newborn health care services and support systems in Buikwe District, Uganda: A qualitative study
Source: PLoS One. 2021 Dec 16;16(12):e0261414. doi: 10.1371/journal.pone.0261414 (PMC8675744; doi:10.1371/journal.pone.0261414)
Supplement: S2 File — (DOCX) [file pone.0261414.s002.docx]

**S2 File. Interview guide for focus group discussions and mothers**

Introduction (by the moderator):

Breastfeeding is quite common in Uganda and has many benefits both for the mother and baby. New mothers are often met with many advices and recommendations from family, friends and health workers. You have been invited to take part in this study to reflect upon the topics of breastfeeding, colostrum and pre-lacteals and to share your positive and negative experiences in the past month since you had the baby.

(Do an introductory round of the participants where they can tell a little bit about themselves and be given a number for identification.)

Initiation of breastfeeding

1. Can you tell me about where you gave birth?

- Did you have any options?
- What made you choose this place?
- Will you go to the same place next time?

1. What happened after birth?

- Did you stay with your baby?
- Was it taken to another room?
- What does the baby need the first day?

1. What can you tell me about putting the baby to the breast the first time?

- How soon after birth did you start breastfeeding the baby?

1. Did you get any information about breastfeeding?

From whom?

- When?
- What information were you given?

1. Have you heard of skin-to-skin contact or kangaroo care?

(if yes: can you tell me about it?)

1. Did you experience any problems when you started to breastfeed?

- (sore nipples, too much/too little milk, infection in the breast etc.)
- If yes, who did you ask for help?
- Did you get the help you needed from the place/person you asked?

Colostrum

1. How will you describe the very first milk?

- Do you feed it to your baby?

1. Have you heard of any reason why this milk should not be given?

- If yes, what are the reasons?

1. Can you tell me any good things about this milk?

Pre-lacteals

1. What happens if the milk does not come into the breast the first day?

- Do you give other foods to the baby?

1. What food is it common to give the baby other than breastmilk?
2. What are the recommendations from health workers/TBA on this issue?
3. Have you been given advice about pre-lacteals from other women in the family/village?
