# Supplementary material for: Women’s experiences of maternal and newborn health care services and support systems in Buikwe District, Uganda: A qualitative study
Source: PLoS One. 2021 Dec 16;16(12):e0261414. doi: 10.1371/journal.pone.0261414 (PMC8675744; doi:10.1371/journal.pone.0261414)
Supplement: Attachment — Submitted filename: Peer review_PONE-D-21-14606.pdf [file pone.0261414.s003.pdf]

Peer review: PONE-D-21-14606

Overall comments:

This is an important piece of work that is addressing a key global health priority. However, the themes reported on maternal experiences of health care are very similar to what has been found in other contexts in sub-Saharan Africa, so it is ever so important that the authors draw out what their key messages are and what this adds to the literature. They also need to link it better to neonatal outcomes as they have stated in the title. Unfortunately, most sections are not written at the level required for a publishable manuscript. Some of the sections are very long and lack focus (e.g. Introduction and Results). The Methods section needs to be reorganised and, in some sections, explained in more depth. The whole manuscript would benefit from a review of the grammar.

Title: *Women's access to and use of maternal health care and home support systems to*

reduce neonatal deaths in Buikwe district, Uganda: a qualitative study

I appreciate the complexity of what was being explore in this study but suggest the title captures this work more succinctly. For example:

Women's experiences of maternal and newborn health care services in Buikwe district, Uganda: a qualitative study

Abstract

Background: *This study aimed <u>at describing</u> maternal support systems* – revise to This study aimed to describe….

Methods and findings:

Data Management is missing.

Line 73***…****prohibited women <u>in</u> receiving optimal* – replace in with from.

Line 38 Unclear how *" Postnatal follow-ups were found unsatisfactory."* This needs to be explained a bit more in the Abstract as it is linked to a recommendation in the Conclusion.

Please review the grammar in the Abstract.

Introduction

This section needs to be a lot more succinct and linked to the aims of the study. As written, it is too long. Some of this content can be moved to the Discussion.

Some specific points:

Line 47 … *2030 if today's trend continues*- revise to: if the current trend continues.

Line 51-53…. *For the mothers it is also important to have someone to trust and rely on during pregnancy, time of childbirth and the following post-partum period, and by such providing an optimal start in life for the newborns (3).* So that…please expand on this.

Methods

Line 108 …*individual interviews* – I am not clear what individual interviews are because in qualitative research, interviews are conducted between a researcher and a participant and not a group. Please clarify.

Line 108…*key-informants*- do you mean key informant interviews?

Line 113- *A summary of methods is given below:* please delete this as it doesn't provide any additional

Line 127- *Participants were selected purposefully* – based on what characteristics? Please expand. (mentioned in line 149-150)

Line 129- *Sample* – This subtitle should be "Sampling". It would be useful to have a Table linked to this section to summarise the sampling strategy.

Line 176 *Instruments* This should be incorporated at the beginning of the sub-section: Data Collection.

Data Management is missing.

Results

Introductory paragraph needs to be revised as it doesn't lead the reader well into the content of the results. This section is also long so the authors need to decide what the key messages are and re-write this in a logical manner that brings out the key messages.

Discussion

This section of the manuscript is more structured with clearer messages of the relevance of this work in the context of existing literature on the topic and context. However, the focus on maternal experiences doesn't link adequately to neonatal outcomes as stated in the title so I wonder whether the paper should focus on maternal experiences of accessing health care.

Conclusion

No comments
