# Supplementary material for: Women’s experiences of maternal and newborn health care services and support systems in Buikwe District, Uganda: A qualitative study
Source: PLoS One. 2021 Dec 16;16(12):e0261414. doi: 10.1371/journal.pone.0261414 (PMC8675744; doi:10.1371/journal.pone.0261414)
Supplement: Attachment — Submitted filename: Peer review_PONE-D-21-14606_R1.pdf [file pone.0261414.s005.pdf]

Peer review: PONE-D-21-14606_R1

Overall comments:

Thank you for addressing most of my comments. This manuscript reads much better but there are grammatical errors in all sections that need to be addressed, as it cannot be published like this. Other aspects that need to be addressed:

Introduction:

This remains very long and lacks focus. Please limit this to 3-4 paragraphs that really draw the reader to the aims/objectives of the study. Some of the content here can be moved to the Discussion.

Results

The sub-title is now “Results and discussion” which is incorrect. This should just be Results.

The current format that combines results and the discussion for a specific result is not one that I am familiar with. I think this should use the standard format of a Results section then Discussion.

The content in the Results and Discussion seems appropriate. I would like to see how the Discussion is written in one section and not combined with the Results.
