# Supplementary material for: Women’s experiences of maternal and newborn health care services and support systems in Buikwe District, Uganda: A qualitative study
Source: PLoS One. 2021 Dec 16;16(12):e0261414. doi: 10.1371/journal.pone.0261414 (PMC8675744; doi:10.1371/journal.pone.0261414)
Supplement: Attachment — Submitted filename: Response to Reviewers .docx [file pone.0261414.s006.docx]

PONE-D-21-14606R2

Women’s experiences of maternal and newborn health care services and support systems in Buikwe District, Uganda: A qualitative study

Dear Editor and Reviewers at PLOS ONE,

The authors appreciate the constructive and thorough reviews. We have adhered to the suggestion of the third reviewer and the manuscript is now in standard format with Discussion following Results. The manuscript has been professionally proofread to improve the grammar. A point-by-point response letter to the comments suggested by the editors and reviewer is given below. As requested, we have updated the information in the Data Availability statement to comply with PLOS ONE submission guidelines.

Sincerely, on behalf of the authors,

Marte Bodil Roed.

PLOS ONE

**Journal Requirements:**

Please review your reference list to ensure that it is complete and correct. If you have cited papers that have been retracted, please include the rationale for doing so in the manuscript text, or remove these references and replace them with relevant current references. Any changes to the reference list should be mentioned in the rebuttal letter that accompanies your revised manuscript. If you need to cite a retracted article, indicate the article’s retracted status in the References list and also include a citation and full reference for the retraction notice.

The Reference list has been updated and is complete. Some citations and references have been omitted due to editing and reduced length of the paper.

A) Please provide non-author contact information for a data access committee, ethics committee, or other institutional body to which data requests may be sent.

The Data Availability statement now includes the name and e-mail address to the Centre for International Health, University of Bergen, Norway. Reasonable requests for data access may be sent to [post@cih.uib.no](mailto:post@cih.uib.no).

**Reviewers' comments:**

(Peer review: PONE-D-21-14606_R1)

Thank you for addressing most of my comments. This manuscript reads much better but there are grammatical errors in all sections that need to be addressed, as it cannot be published like this.

The manuscript has undergone academic proofreading by a professional company approved and affiliated by the University of Bergen (Semantix). Suggestions and changes have been discussed among the authors and implemented accordingly.

Other aspects that need to be addressed:

Introduction:

This remains very long and lacks focus. Please limit this to 3-4 paragraphs that really draw the reader to the aims/objectives of the study. Some of the content here can be moved to the Discussion.

The first sentence in the abstract and introduction has been re-written.
The introduction section has been reduced to 4 paragraphs and several components have been moved to discussion or omitted. It should now point more clearly towards the objectives of the study.

Results

The sub-title is now “Results and discussion” which is incorrect. This should just be Results.

The sub-titles Results and Discussion are now separated.

The current format that combines results and the discussion for a specific result is not one that I am familiar with. I think this should use the standard format of a Results section then Discussion.

The authors agree that the format used in this paper is unusual. We have considered the various formats and the Results and Discussion sections are now again written in standard format in separate sections. The discussion has been edited and elaborated with contents added from the Introduction section.

The content in the Results and Discussion seems appropriate. I would like to see how the Discussion is written in one section and not combined with the Results.

Thank you for your clear views and frank suggestions. The Result and Discussion sections are now separated.
